# Characteristics, satisfiers, development needs, and barriers to success for early-career academic hospitalists

**DOI:** 10.1186/s12909-022-03356-0

**Published:** 2022-04-13

**Authors:** Shradha A. Kulkarni, Margaret C. Fang, Jeffrey J. Glasheen, Vikas Parekh, Bradley A. Sharpe

**Affiliations:** 1 Division of Hospital Medicine, Department of Medicine, University of California, 521 Parnassus Avenue, Box 0131, San Francisco, CA 94143 USA; 2 Department of Medicine, University of Colorado, Aurora, CO USA; 3 Department of Internal Medicine, University of Michigan, Ann Arbor, MI USA

**Keywords:** Hospital medicine, Faculty development, Medical education, Bedside teaching, Feedback

## Abstract

**Background:**

Academic hospitalists engage in many non-clinical domains. Success in these domains requires support, mentorship, protected time, and networks. To address these non-clinical competencies, faculty development programs have been implemented.

We aim to describe the demographics, job characteristics, satisfiers, and barriers to success of early-career academic hospitalists who attended the Academic Hospitalist Academic (AHA), a professional development conference from 2009 to 2019.

**Methods:**

Survey responses from attendees were evaluated; statistical analyses and linear regression were performed for numerical responses and qualitative coding was performed for textual responses.

**Results:**

A total of 965 hospitalists attended the AHA from 2009 to 2019. Of those, 812 (84%) completed the survey. The mean age of participants was 34 years and the mean time in hospitalist practice was 3.2 years. Most hospitalists were satisfied with their job, and teaching and clinical care were identified as the best parts of the job. The proportion of female hospitalists increased from 42.2% in 2009 to 60% in 2019 (*p* = 0.001). No other demographics or job characteristics significantly changed over the years. Lack of time and confidence in individual skills were the most common barriers identified in both bedside teaching and providing feedback, and providing constructive feedback was an additional challenge identified in giving feedback.

**Conclusions:**

Though early-career hospitalists reported high levels of job satisfaction driven by teaching and clinical care, barriers to success include time constraints and confidence. Awareness of these factors of satisfaction and barriers to success can help shape faculty development curricula for early-career hospitalists.

## Introduction

The academic hospitalist movement began in the late 1990s and has subsequently grown and evolved rapidly [1–3]. As health systems sought improved efficiency and shifted away from outpatient providers managing their own hospitalized patients, the role of a hospitalist was created [2]. Adult hospitalists are physicians most typically trained in family or internal medicine who serve as primary clinicians for hospitalized patients. Hospitalists can also fulfill a variety of other clinical roles such as the co-management of surgical patients, consultation on non-internal medicine hospitalized patients, and evaluation of inter-hospital transfers [ 2]. While the hospitalist movement originated in the United States, similar models have been adapted in other parts of the world including Europe and Asia [3, 4]. In addition to patient care, academic hospitalists, those practicing in academic medical centers, engage in multiple non-clinical career domains, including medical education, quality improvement, leadership, and research. Success in these domains requires support through mentorship, protected time for non-clinical work, and professional networks [5–7]. The career structure of academic hospitalists typically involves advancement through standard institutional designations of Clinical Instructor, Assistant Professor, Associate Professor, and Professor. Early-career hospitalists are generally considered to be those at the Clinical Instructor or Assistant Professor level who would benefit from faculty development programs and mentorship from senior faculty members [8, 9].

To our knowledge, current literature on identifying specific themes for challenges that early-career academic hospitalists perceive in their clinical and non-clinical roles is limited. Small survey reports have identified a lack of protected time and a need for additional mentorship as barriers to pursuing scholarly work outside of patient care [5–7, 10, 11]. The limited guidance for non-clinical pursuits may have led to a degree of ambivalence for these endeavors [8]. Whereas perceptions from early-career hospitalists are relatively unknown, senior hospitalist leadership has consistently noted a need for improving mentorship and career development [6, 12].

To address these barriers to success in non-clinical competencies, some institutions have implemented faculty development programs, which have led to improved work satisfaction and academic output [13–15].

In an attempt to build a national faculty development and mentorship program, the Academic Hospitalist Academy Level 1 (AHA) was created in 2009 with the goal of enhancing education and professional development skills to address needs identified by a national survey of leaders of academic hospitalist groups.

The AHA is advertised to hundreds of hospital medicine groups. Attendance is voluntary and highly recommended for early-career hospitalists by senior mentors. Faculty are often provided discretionary funding to support attendance of the conference, and attendees represent numerous diverse hospital medicine programs in the United States. The AHA has continued annually since inception, and we aim to describe the demographics, job characteristics, satisfiers, and barriers to success of the early-career academic hospitalists who have attended the conference to characterize the evolution and current status of the field.

## Methods

In 2009, the Society of Hospital Medicine (SHM), the Society of General Internal Medicine (SGIM), and the Association of Chiefs and Leaders of General Internal Medicine (ACLGIM) launched the AHA, an annual national conference designed to provide hospitalists professional development skills in non-clinical domains such as teaching, feedback, quality improvement, and leadership. The conference is four days and consists of interactive presentations, small-group exercises, and skill-building breakout sessions. There is 1:10 “faculty” (e.g. academic hospitalist leaders) to “attendee” (e.g. early-career academic hospitalists) ratio.

AHA Level 1 is specifically geared towards early-career academic hospitalists and is the focus of our paper (and all reported data are from AHA Level 1). There is also an AHA Level 2, which is intended for mid-career hospitalists. For the purposes of this paper, AHA refers to AHA Level 1.

As part of the AHA registration process from 2009 to 2019, the enrollees received an electronic link to an anonymized, voluntary survey to enter information on demographics, career satisfaction, and barriers to success. Demographic survey questions included age, gender, years of experience, and academic rank. From the demographic information provided, the average age and years of experience of participants was calculated and 95% confidence intervals (CIs) were determined. Additionally, proportions for gender and academic rank were calculated. Regarding career satisfaction, questions included predetermined Likert scales on topics including job satisfaction, proportion of clinical time on direct care or teaching service, and percent protected time. A linear regression to evaluate changes over time with respect to gender and job satisfaction was performed.

Several free-text questions asked attendees to reflect on barriers to success. Two domains that were specifically emphasized were bedside teaching and feedback, as bedside teaching is underutilized yet valued in academia, and providing feedback has been identified as both valuable and challenging [16–22]. The questions for these domains were as follows:

1. List 3 barriers to being an effective bedside teacher (Table 3 in Results)
2. List 3 challenges you encounter in giving feedback to learners (Table 4 in Results)

Responses were reviewed by one of the authors (SAK), who defined coding categories using content analysis to capture the described barriers [23, 24]. Qualitative codes were then quantified (by counting the numeric frequency of each coding category) to understand the prevalence of each barrier. To confirm that the categorization was accurate and consistent, 10% of free-response answers (492 answers) were independently adjudicated by a second independent coder. All survey responses were de-identified, and this study was deemed exempt by the Institutional Review Board of the University of California San Francisco given the lack of identifying information.

## Results

**Fig. 1:**
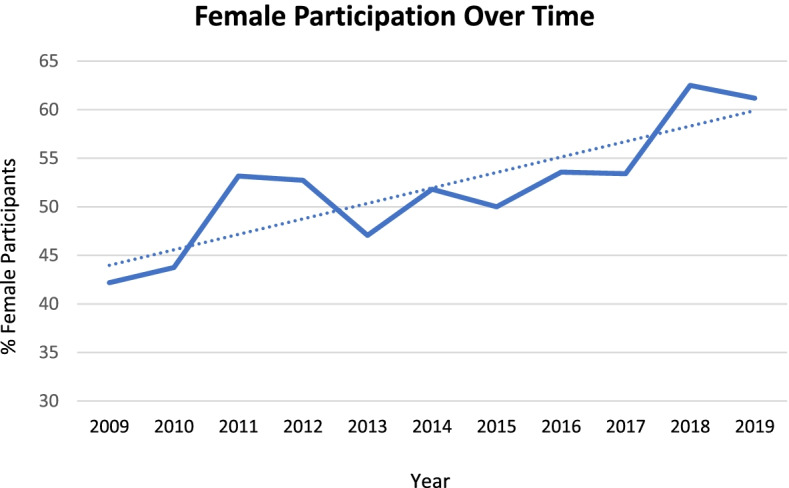
Female participation in AHA. *dotted line indicates linear regression trend line

A total of 965 hospitalists attended the AHA from 2009 to 2019. Of those, 812 (84%) completed at least a portion of the survey. The mean age of participants was 34 years (95% CI 25–44 years) and the mean time in hospitalist practice was 3.2 years [0–9.4 years] (Table 1). The most common academic rank was Assistant Professor (59.9%) or Clinical Instructor (35.4%), while Associate Professor and Professor together comprised less than 3% of participants, indicating the vast majority of participants were early-career hospitalists. Linear regression showed that the proportion of female participants increased from 42.2% in 2009 to 60% in 2018 and 2019 (*p*-value for trend = 0.001) (Fig. 1). Most hospitalists were somewhat (50.1%) or very (36.6%) satisfied with their job; linear regression did not show a change in satisfaction over time (p-value for trend = 0.96). Teaching and clinical care were consistently identified as the best parts of the job (56.0 and 31.1%, respectively, Table 2).

**Table 1:** Hospitalist demographics, AHA 2009–2019

Characteristic	AHA attendees
Age (years) (*n* = 807)	34.4 ± 4.7
Clinical experience (years) (*n* = 800)	3.2 ± 3.1
Female gender (*n* = 807) [n, %]	428 (53.0)
Involved in QI (*n* = 762) [n, %]	369 (48.4)
Participated in event review (*n* = 758) [n, %]	341 (45.0)
Has a mentor (*n* = 753) [n, %]	333 (44.2)
Has an external reference (*n* = 749) [n, %]	304 (40.6)
Attended SGIM or SHM conference in last 1 year (*n* = 700) [n, %]	205 (29.3)
Presented at national meeting in last 2 years (*n* = 703) [n, %]	182 (25.9)
Academic Title (*n* = 760) [n, %]	
Lecturer	16 (2.1)
Instructor	269 (35.4)
**Assistant Professor**	**455 (59.9)**
Associate Professor	19 (2.5)
Professor	1 (0.1)

**Table 2:** Attendees’ job characteristics, AHA 2009–2019

Question Stem	Response Choice	n (%)
Favorite part of job (*n* = 705)	**Teaching**	**395 (56.0)**
	Clinical Care	219 (31.1)
	QI/Safety	45 (6.4)
	Weekends Off	19 (2.7)
	Co-Management	6 (0.8)
	Writing Papers	1 (0.1)
	Emails	1 (0.1)
	Other	19 (2.7)
Job satisfaction (*n* = 808)		
	Not satisfied	25 (3.1)
	Somewhat unsatisfied	82 (10.1)
	**Somewhat satisfied**	**405 (50.1)**
	Very satisfied	296 (36.6)
Percent protected time (*n* = 743)		
(not asked in 2009)	**0%**	**312 (42.0)**
	1–10%	200 (26.9)
	11–25%	166 (22.3)
	26–49%	55 (7.4)
	50% or more	9 (1.2)
Percent of time on direct care (*n* = 807)		
	0–24%	111 (13.8)
	25–49%	126 (15.6)
	50–74%	204 (25.3)
	**75–100%**	**367 (45.5)**
Weeks as attending (*n* = 599)		
(not asked 2009–2011)	0–4 weeks	105 (17.5)
	5–8 weeks	141 (23.5)
	9–12 weeks	99 (16.5)
	13–16 weeks	75 (12.5)
	**16 weeks or more**	**179 (29.9)**

Improving teaching skills was the most common primary conference goal listed by attendees (48.9%), followed by networking, promotion, scholarship, and quality improvement. Seventy-eight percent (78%) of attendees listed improving teaching as one of their top three goals in attending the Academy. Over 91% of participants reported having less than 25% protected time (time when not on a clinical patient care service), with 42% of participants reporting having zero protected time (Table 2). Nearly half (45.5%) of participants cared for patients on a direct-care service for at least 75% of their clinical time. The distribution for weeks as an attending on wards was heterogeneous: 41% had 0–8 weeks, 29% had 9–16 weeks, and 30% had more than 16 weeks as a teaching attending. The average age, years of hospitalist practice, academic rank, amount of protected time, direct care service, and time attending on teaching wards did not significantly change over time.

Of the 812 hospitalists who completed the survey, 702 (86.4%) filled out the free response portion and identified barriers to bedside teaching (Table 3). Eight distinct categories were able to be identified. Lack of time was the most commonly reported barrier to bedside teaching, reported by 86.8% of hospitalists, and named as the leading barrier by the majority of hospitalists (53.7%). Free-text quotations from attendees indicated that “time constraints, particularly with the institution emphasizing early discharges and turnover” and the “conflict on time spent at bedside with time reviewing data and discussion of the plan” made bedside teaching challenging. Other commonly identified barriers included a lack of confidence in individual skills (74.6%), patient preferences (26.6%), and lack of perceived interest from learners (19.8%). Specific reflections included “personal lack of knowledge,” “[dis] comfort of my physical exam skills,” and challenges in “managing simultaneously to teach learners at multiple levels.”

**Table 3:** Barriers to bedside teaching, AHA 2009–2019

Barrier [n,%]	#1 Barrier^**a**^ (***n*** = 702)	#2 Barrier (***n*** = 646)	#3 Barrier (***n*** = 533)	Top 3 Barrier (% of 702)
Time	377 (53.7)	142	90	609 (86.8)
Personal skills/confidence	154 (21.9)	193	177	524 (74.6)
Patient preferences	34 (4.8)	76	77	187 (26.6)
Patient complexity/volume	32 (4.5)	37	18	87 (12.4)
Space	26 (3.7)	35	33	94 (13.4)
Including all learners	22 (3.1)	52	43	117 (16.7)
Distractions	22 (3.1)	26	19	67 (9.5)
Resident interest	21 (3.0)	69	49	139 (19.8)
Other	14 (2.0)	16	27	57 (8.1)

^a^ Participants could list up to 3 barriers, hence the variation in n for each barrier

Of the 812 hospitalists who completed the survey, 685 (84.4%) identified barriers to providing feedback (Table 4). Ten distinct coding categories were identified as barriers to providing feedback. Categories included a lack of confidence in individual skills, listed as a barrier by 80.2% of hospitalists who responded, followed by hesitancy to give negative feedback (43.9%), lack of time (38.1%), lack of sufficient exposure to trainees (23.8%), and lack of perceived receptiveness by learners (23.4%). Lack of confidence in individual skills was named as the leading barrier by 25.6% of hospitalists, while hesitancy to give negative feedback was the leading barrier for 25.3% of hospitalists. Specific quotations from hospitalists included: “being honest when there is an area of concern” and “[providing] negative feedback when the learner doesn’t seem motivated.”

**Table 4:** Barriers to providing feedback, AHA 2009–2019

Barrier [n, %]	#1 Barrier (***n*** = 685)	#2 Barrier (***n*** = 602)	#3 Barrier (***n*** = 457)	Top 3 Barrier (% of 685)
Lack of personal skills/confidence	175 (25.6)	195	179	549 (80.2)
Hesitant to give negative feedback	173 (25.3)	78	42	293 (43.9)
Lack of time	110 (16.1)	91	60	261 (38.1)
Learners’ lack of receptiveness	64 (9.3)	59	37	160 (23.4)
Lack of exposure to trainees	55 (8.0)	65	43	163 (23.8)
Lack of specific examples	47 (6.9)	47	24	118 (17.2)
Fear of repercussions for negative feedback	25 (3.6)	20	13	58 (8.5)
Unknown expectations	14 (2.0)	22	17	53 (7.7)
Stellar learners	12 (1.8)	12	12	36 (5.3)
Space	5 (0.7)	10	18	33 (4.8)
Distractions/Other	5 (0.7)	4	12	21 (3.1)

## Discussion

Most hospitalists who enrolled in AHA were relatively young academic hospitalists in the first 5 years of their job and this, along with academic rank, protected non-clinical time, and clinical time with learners did not change over the 11 years of the AHA. However, enrollment by female faculty increased significantly and women comprised the majority of participants in recent years. This aligns with findings by the Association of American Medical Colleges, which has noted a consistently increasing proportion of female physicians over the past decade [25].

Attendees reported high levels of job satisfaction and this finding was consistent over time. Prior studies have similarly noted a generally high level of job satisfaction among hospitalists [26, 27]. Bedside teaching was consistently identified as the main contributor to job satisfaction. Academic hospitalists have indicated that non-clinical aspects, such as teaching, provide fulfillment and are an important contributor to job satisfaction [8, 11]. Improvement in teaching skills was the primary goal for hospitalists attending the AHA, which indicates a priority for early-career hospitalists to optimize their teaching skills. Importantly, as clinical time with learners may decrease in the future as the clinical demands of hospitalists and residents evolve, academic hospitalist groups will need to consider methods to continue engaging their faculty in meaningful ways [28].

Within the domain of bedside teaching, we identified numerous consistent barriers to bedside teaching and the provision of feedback. Lack of confidence and time were two prevalent themes. Lack of confidence was identified as a significant barrier and may speak to the limited experience (both in terms of years of practice and clinical time on teaching wards) of the attendees. Lack of time was cited as a significant limiting factor in both bedside teaching and giving feedback, though it was identified more consistently as a barrier to bedside teaching. This may be due to the innate challenge of balancing bedside teaching with the need to complete time-sensitive clinical care tasks.

In addition to bedside teaching, clinical care was the second leading contributor to job satisfaction. Similar perceptions have been noted in prior studies [11, 26]. This indicates that patient care remains valued and provides an important source of fulfillment for early-career academic hospitalists. However, criteria for promotion at academic institutions most often include non-clinical activities such as research and scholarship. Though protected time for these endeavors is not requisite, it is highly correlated with productivity. Our study found that the vast majority of this early-career academic hospitalist cohort had less than 25% of their time protected for academic work and this likely contributes to the relatively low academic productivity of academic hospitalists [29, 30].

Prior data suggests that faculty development curricula for junior faculty are beneficial, and we advocate for continued support of such programs [13, 31]. Additionally, our findings can serve as a blueprint for developing sessions focused on bedside teaching and feedback. For example, a faculty development session on feedback could include a focus on the barriers identified such as time efficiency, providing constructive feedback, and perceived lack of confidence.

Finally, our finding that the job characteristics of academic hospitalists have not changed over the years highlights a potential area for improvement in the field. Academic hospitalist groups tend to focus on quality- and systems-related initiatives, rather than research in new clinical approaches. This lack of research may limit the advancement of the field [32]. Increasing awareness of and emphasis on such scientific inquiry should be considered.

This study has several limitations. While our evaluation of demographics and job characteristics over time provides the descriptive insights noted above, there are likely additional underlying confounding factors that have impacted academic hospitalists’ job descriptions over the years. These may include factors that are not standardized and vary between institutions, such as clinical workload and compensation structure. Our findings are also specific to academic hospitalists and may not be fully applicable to hospitalists practicing in settings which do not involve significant expectations of academic and non-clinical productivity.

Additionally, though we were able to survey nearly 1000 hospitalists with an over 80% response rate, it is difficult to determine whether the AHA attendees provide an adequate representation of all early-career academic hospitalists. By virtue of attending an academic conference, AHA attendees may represent a phenotype of hospitalist who is more focused on improving teaching and non-clinical skills than academic hospitalists who did not attend or non-academic hospitalists.

## Conclusion

Over the 11 years of the Academic Hospitalist Academy, attendees consistently reported high levels of job satisfaction driven by teaching and clinical care. Still, early-career academic hospitalists noted significant development needs. Improving bedside teaching skills was the most common motivation to attend the conference. Lack of confidence and time constraints were identified as challenges to effectively teaching at the bedside and providing feedback. Awareness of the drivers of satisfaction and barriers to success can help shape faculty development needs for future early-career hospitalists.

## Data Availability

The datasets generated and/or analysed during the current study are not publicly available as they were obtained anonymously from a faculty development conference, but are available from the corresponding author on reasonable request.

## Abbreviations

AHA: Academic Hospitalist Academy
SHM: Society of Hospital Medicine
SGIM: Society of General Internal Medicine
ACLGIM: Association of Chiefs and Leaders of General Internal Medicine
CI: Confidence interval
